# Hexagonal-to-Monoclinic Phase-Modulated HAp Nanofibers for Enhanced Piezoelectric and Biocompatible Performance

**DOI:** 10.3390/biom16030385

**Published:** 2026-03-04

**Authors:** Karime Carrera-Gutiérrez, Estefania Venegas-Contreras, Miguel Márquez-Torres, Marco Antonio Ruiz-Esparza-Rodríguez, Yasmin Esqueda-Barrón, Roberto Gomez-Batres, Irene Leal-Berumen, Jorge Noé Díaz de León, Juan José Gervacio-Arciniega, Guillermo Herrera-Pérez, Victor Manuel Orozco-Carmona, Gabriel Rojas-George

**Affiliations:** 1 Centro de Investigación en Materiales Avanzados (CIMAV), Miguel de Cervantes 120, Chihuahua 31109, Mexico; karime.carrera@cimav.edu.mx (K.C.-G.); estefania.venegas@cimav.edu.mx (E.V.-C.); miguel.marquez@cimav.edu.mx (M.M.-T.); marco.ruiz@cimav.edu.mx (M.A.R.-E.-R.); 2 Centro de Investigación Científica y Educación Superior de Ensenada-CICESE, Ensenada 22860, Mexico; yesqueda@ens.cnyn.unam.mx; 3 Facultad de Medicina y Ciencia Biomédicas, Universidad Autónoma de Chihuahua, Circuito Universitario 31109, Campus UACH II, Chihuahua 31125, Mexico; rgomezb@uach.mx (R.G.-B.); ileal@uach.mx (I.L.-B.); 4 Centro de Nanociencias y Nanotecnología, Universidad Nacional Autónoma de México, Km. 107 Carretera Tijuana-Ensenada, Ensenada 22800, Mexico; noejd@ens.cnyn.unam.mx; 5 SECIHTI-Facultad de Ciencias Físico Matemáticas, Benemérita Universidad Autónoma de Puebla, Puebla 72570, Mexico; jjgervacio@fcfm.buap.mx; 6 SECIHTI-Centro de Investigación en Materiales Avanzados (CIMAV), Miguel de Cervantes 120, Chihuahua 31109, Mexico; guillermo.herrera@cimav.edu.mx

**Keywords:** monoclinic HAp, piezoelectric, nanofibers, Rietveld refinement, TEM, AFM, cell viability and cytotoxicity

## Abstract

In the present manuscript, the influence of reaction time on the hexagonal-to-monoclinic phase transition in hydroxyapatite (HAp) nanofibers synthesized via a low-temperature modified hydrothermal method at 100 °C is investigated. The resulting nanofibers were highly crystalline and stoichiometric, with a Ca/P ratio of approximately 1.67. Comprehensive structural and functional characterization, combining X-ray diffraction with Rietveld refinement, Fourier-transform infrared spectroscopy (FTIR), Raman spectroscopy, transmission electron microscopy (TEM), and resonance-tracking piezoresponse force microscopy (RT-PFM), was employed to elucidate the role of the non-centrosymmetric monoclinic P2_1_/b phase in governing HAp’s structural and piezoelectric properties. The analyses indicated a time-dependent phase evolution from hexagonal (P6_3_/m) to monoclinic (P2_1_/b), with exclusive formation of the hexagonal phase at 6 h and a clearly dominant monoclinic fraction (73.56%) after 24 h. Nanofibers synthesized for 48 h comprised approximately 98% monoclinic HAp and exhibited elongated morphologies with an average length of 354.82 nm and diameter of 45 nm. RT-PFM measurements confirmed a pronounced piezoelectric response associated with the monoclinic phase, yielding an effective piezoelectric coefficient (*d*_eff_) of 19.85 pm/V. In vitro MTT assays demonstrated that the high monoclinic content did not compromise biocompatibility, as cell viability and cytotoxicity met the requirements of ISO 10993 and ASTM F895 standards. These findings offer new insights into how monoclinic ordering governs the piezoelectric behavior of HAp and suggest a promising strategy for enhancing its performance in biomedical applications.

## 1. Introduction

Bone tissue engineering (BTE) increasingly relies on advanced biomaterials that not only provide mechanical support but also actively stimulate bone regeneration [1,2]. In this context, piezoelectric biomaterials have garnered significant interest due to their ability to convert mechanically induced deformations (ubiquitous in bone physiology) into electrical signals that enhance osteogenic activity. Native bone itself exhibits a characteristic piezoelectric response: mechanical loading generates an electrical potential that modulates cellular behavior in accordance with Wolff’s law [3,4,5]. Reported longitudinal piezoelectric coefficients (d_33_) for bone typically range from 0.5 to 2.3 pC/N [6,7,8], while the converse piezoelectric coefficient falls between 0.1 and 10 pm/V [9,10].

These electromechanical properties underscore the need for biomaterials that faithfully replicate the intrinsic electromechanical behavior of native bone by integrating compatible structural and functional characteristics into a single system. Such functionality is particularly valuable in implant coatings and composite scaffolds, where surface charge can significantly enhance cell adhesion, proliferation, and differentiation [11,12]. Moreover, elongated hydroxyapatite (HAp) nanostructures (including nanorods, nanofibers, nanobelts, and nanowires) demonstrate superior biological performance due to their high surface area, enhanced protein adsorption, and improved osteogenic capacity [13,14], making them highly attractive for scaffold design.

Hydroxyapatite (HAp, Ca_10_(PO_4_)_6_(OH)_2_) is widely employed in BTE owing to its excellent biocompatibility, bioactivity, and chemical similarity to the mineral phase of bone [15]. HAp exists in two closely related polymorphs: hexagonal (P6_3_/m; lattice parameters a = b ≈ 9.417 Å, c ≈ 6.875 Å, α = β = 90°, γ = 120°) and monoclinic (P2_1_/b; a ≈ 9.480 Å, b ≈ 18.960 Å, c ≈ 6.830 Å, α = β = 90°, γ = 120°) [16]. In the monoclinic phase, cooperative ordering of OH^−^ ions gives rise to non-centrosymmetric domains, a structural feature essential for piezoelectricity [17]. This long-range OH^−^ ordering provides the fundamental basis for the piezoelectric response observed in monoclinic HAp [18].

However, synthetic HAp is most commonly reported in the hexagonal form, as the monoclinic phase typically forms only at high temperatures (~1000 °C) [19,20,21]. The monoclinic fraction is highly sensitive to synthesis conditions, including temperature, pH, reaction time, and precursor chemistry, all of which influence crystal growth kinetics, stoichiometry, and defect concentration [22,23]. Stoichiometric HAp (Ca/P = 1.67) favors monoclinic ordering, whereas Ca-deficient hydroxyapatite (Ca-D HAp) or carbonate-substituted hydroxyapatite (C-HAp) stabilize the centrosymmetric hexagonal phase [24]. Consistent with this symmetry dependence, bulk hexagonal HAp exhibits low d_33_ values (0.1–1.5 pC/N), typically attributed to defect-induced or nanoscale symmetry breaking rather than intrinsic piezoelectricity [25,26,27].

Nevertheless, Han et al. (2024) demonstrated that hexagonal HAp nanorods can exhibit a remarkably high piezoelectric response (d_33_ ≈ 3.29 pm/V) when structural gradient effects induce local asymmetry [28]. In contrast, monoclinic HAp generally displays higher and more consistent piezoelectric coefficients (typically around 8 pm/V) as reported in studies on structurally ordered or monoclinic-rich ceramics [29,30,31]. Notably, Pérez-Solís et al. (2018) reported an exceptionally high piezoelectric coefficient of approximately 10 pm/V in pellets fabricated from HAp powders synthesized via an ultrasound-assisted sol–gel route, which contained ~85% monoclinic phase [32]. This finding underscores that a predominantly monoclinic structure can substantially enhance the electromechanical performance of HAp.

Since intrinsic piezoelectricity requires a non-centrosymmetric crystal structure [33], current research efforts are increasingly focused on stabilizing the monoclinic P2_1_/b polymorph—particularly in elongated nanostructures that mimic the morphology of natural bone [34,35]. In this regard, Sánchez-Campos et al. (2021) successfully synthesized ultrathin HAp nanorods (diameter: 29.54 nm; length: ~106.6 nm) with 90–95% monoclinic content under mild microwave-assisted hydrothermal conditions by precise pH control [36]. However, that study did not assess the piezoelectric response, leaving the electromechanical properties of these highly monoclinic nanorods uncharacterized.

Distinguishing the hexagonal-to-monoclinic phase transition in HAp remains challenging due to the striking similarity in their lattice parameters, which leads to significant overlap in X-ray diffraction (XRD) peaks [37]. Moreover, synthesis often results in secondary calcium phosphate phases whose diffraction signatures closely resemble those of HAp [38]. Consequently, reliable identification of the monoclinic phase necessitates complementary analytical techniques. Rietveld refinement of XRD data can resolve subtle crystallographic differences between the two polymorphs, while Fourier-transform infrared (FTIR) and Raman spectroscopies (both highly sensitive to local symmetry) detect monoclinic ordering through the sharpening or splitting of phosphate vibrational modes and distinct changes in OH^−^ stretching signals [39].

To the best of the authors’ knowledge, while much of the current research on hydroxyapatite aims to stabilize the monoclinic phase to enhance piezoelectricity for bone regeneration applications, its biological implications, particularly in highly crystalline, phase-pure nanostructures, remain poorly understood. In this work, we report the low-temperature synthesis of highly crystalline, elongated HAp nanofibers via a modified hydrothermal method, effectively avoiding the formation of secondary calcium phosphate phases. By systematically varying reaction time, is monitored the hexagonal-to-monoclinic phase evolution using a multimodal characterization approach (XRD with Rietveld refinement, nanobeam diffraction [NBD], FTIR, Raman, EDXS, and XPS). Resonance tracking piezoresponse force microscopy (RT-PFM) revealed that increasing monoclinic content directly enhances piezoelectric activity, while in vitro MTT assays confirmed that high monoclinic content does not compromise biocompatibility. These findings demonstrate that monoclinic HAp can significantly improve electromechanical functionality without adverse effects on biological performance, highlighting its strong potential for advanced bone tissue engineering applications.

## 2. Materials and Methods

Hydroxyapatite (HAp) powders were synthesized via a modified hydrothermal method [40]. To investigate the influence of reaction time on the hexagonal-to-monoclinic phase transition, five samples were prepared with hydrothermal durations of 6, 12, 24, and 48 h, designated as S6, S12, S24, S36, and S48, respectively. Calcium nitrate tetrahydrate [Ca(NO_3_)_2_·4H_2_O, 99% purity, Cat. No. A16645, Alfa Aesar, Walthman, MA, USA] and diammonium hydrogen phosphate [(NH_4_)_2_HPO_4_, 98% purity, Cat. No. 11597, Alfa Aesar, Walthman, MA, USA] served as the calcium and phosphorus precursors, respectively. Aqueous solutions (0.2 M) of both precursors were prepared to achieve a stoichiometric Ca/P molar ratio of 1.67.

The Ca(NO_3_)_2_·4H_2_O solution was first heated to 100 °C under magnetic stirring in a three-neck flask. The (NH_4_)_2_HPO_4_ solution was then added dropwise into the heated calcium solution. The resulting mixture was maintained at 100 °C under constant stirring for the designated reaction time. Upon completion, the suspension was transferred to an Erlenmeyer flask and allowed to cool to room temperature under static conditions until a white precipitate formed. The precipitate was collected by filtration, thoroughly washed with deionized water, dried at 50 °C for 2 h, and finally ground in an agate mortar for 30 min to obtain fine HAp powder.

### 2.1. Structural and Compositional Characterization

The crystalline phase composition was analyzed by X-ray diffraction (XRD) using a PANalytical X’Pert PRO diffractometer (Malvern Panalytical, Almelo, The Netherlands) equipped with an X’Celerator detector and CuKα radiation (λ = 1.5405 Å). Diffraction patterns were recorded at room temperature in Bragg–Brentano geometry over a 2θ range of 20–80°, with a step size of 0.017° and a counting time of 500 s per step. Rietveld refinement [41] was performed using the FullProf Suite (version April 2025) [42] to quantify the relative weight percentages of hexagonal (P6_3_/m) and monoclinic (P2_1_/b) HAp phases and to determine their lattice parameters.

For sample S6, the refinement model included only the hexagonal phase, based on the structural data reported by Yacoubi et al. (2017) [43]. For samples S12, S24, S36, and S48, a biphasic model incorporating both hexagonal and monoclinic phases was employed. Initial atomic displacement parameters (U) for the monoclinic phase were taken from Pérez-Solís et al. [32]. The refined structural models were visualized using VESTA software (version 3.90.5a) [44].

Functional groups and local symmetry were probed by Fourier-transform infrared spectroscopy (FTIR) on a PerkinElmer Spectrum GX FTIR system over the wavenumber range of 4000–400 cm^−1^. Complementary Raman spectroscopy was performed using a Horiba XploRA confocal Raman microscope (HORIBA Advanced Techno, Co., Ltd., Kisshoin Minami-ku Kyoto, Japan) with laser excitation at 638 nm (red) and 785 nm (near-infrared).

### 2.2. Morphological and Microstructural Analysis

Morphology and nanostructure were examined by transmission electron microscopy (TEM) using a JEOL JEM-2200FS + Cs instrument (JEOL, Tokyo, Japan) operated at 200 kV, with a point-to-point resolution of 1.9 Å. For improved statistical analysis of HAp nanofiber size distributions, a total of 100 nanofibers (*n* = 100) were analyzed per sample. For this purpose, five TEM grids (300-mesh copper grids, PELCO^®^ TEM Grids, Ted Pella Inc., Redding, CA, USA) were prepared and analyzed from dispersions of 0.5 mg/mL of each sample in deionized water. Nanofiber dimensions (length and diameter) were measured from TEM micrographs using ImageJ software (version 1.53t) following calibration with high-resolution TEM images [45]. The chemical composition was analyzed by energy-dispersive X-ray spectroscopy (EDXS) coupled to the TEM. Additionally, X-ray photoelectron spectroscopy (XPS) was carried out using a SPECS system equipped with a PHOIBOS WAL concentric hemispherical analyzer and an Al Kα X-ray source (1486.61 eV). Survey and high-resolution spectra were processed with CasaXPS software (version 2.3.24) [46] after Shirley background subtraction. Quantitative elemental analysis was performed using Scofield photoionization cross-section sensitivity factors.

### 2.3. Piezoelectric Characterization

Piezoelectric properties were evaluated by resonance tracking piezoresponse force microscopy (RT-PFM), using an atomic force microscope (Park Systems XE7, Albany, NY, USA) integrated with a Stanford Research SR865A lock-in amplifier and controlled via a custom LabVIEW^®^ 2018 program (National Instruments, Austin, TX, USA) [47]. Measurements were conducted using a 450-μm-long Pt/Ir-coated conductive cantilever (BudgetSensors, Sofia, Bulgaria) with a spring constant of 0.2 N/m and a resonance frequency of ~15 kHz. Powder samples were mounted on platinum substrates (Bayville Chemical Supply Company, Inc., Smithtown, NY, USA) using silver paint (SPI Supplies, Dotite D-550 Silver Colloid, #05003-AB, West Chester, PA, USA) to ensure electrical contact and mechanical stability. To minimize electrostatic artifacts, DC bias was kept “off” during local polarization hysteresis loop acquisition [48,49]. A lead zirconate titanate (PZT) ceramic was used as a calibration reference. The effective piezoelectric coefficient (d_eff_) was determined via RT-PFM: the AC voltage was swept from 0 to 2 V in 0.1 V steps at a fixed sample location while tracking the resonant PFM response. The piezoresponse amplitude signal, deconvoluted by the lock-in amplifier, was recorded using the LabVIEW (version 2025 Q3) program. A linear relationship between the applied voltage and the piezoresponse was observed for both HAp and PZT. RT-PFM measurements were performed on three different nanofibers to calculate an average effective piezoelectric coefficient (*d*_eff_) for each powder sample. The slope of the voltage–piezoresponse relationship for HAp, normalized against the PZT reference, enabled the calculation of *d*_eff_ using the established calibration protocol [50].

(1) $$d_{33HAp}=\frac{m_{HAp}(d_{33PZT})}{m_{PZT}}$$

where m_HAp_ and m_PZT_ are the slopes of the linear piezoresponse–voltage curves for HAp and PZT, respectively. The reference PZT sample was assigned a d_33_ value of 55 pm/V, consistent with literature values obtained via Switching Spectroscopy Piezoresponse Force Microscopy (SS-PFM) in an Atomic Force Microscope (AFM) model Smena-A (NT-MDT Spectrum Instruments, Zelenograd, Moscow, Russia) [51].

Topography, amplitude, and phase images were acquired using Piezoresponse Force Microscopy (PFM) in the PinPoint™ nanomechanical mapping mode [52], which minimizes sample deformation and is particularly suitable for studying the piezo-ferroelectric response of soft or fragile materials such as HAp powders. Measurements were performed on 256 × 256 pixel grids with an AC voltage of 1 V applied at a frequency of 200 kHz, following the experimental protocol described by Pérez Solís et al. [32]. This approach enabled the simultaneous acquisition of topography, particle boundaries, and local electromechanical (ferroelectric like) domain information, even for unconsolidated powder samples.

### 2.4. Biocompatibility Assessment

Biocompatibility was evaluated in vitro through cytotoxicity and cell viability assays using the MTT assay, which measures the reduction of 3-(4,5-dimethylthiazol-2-yl)-2,5-diphenyltetrazolium bromide (MTT) to insoluble formazan crystals by mitochondrial dehydrogenases in metabolically active cells [53].

For indirect-contact cytotoxicity testing (per ISO 10993-5 [54]), HAp powder samples were immersed in RPMI-1640 medium (Gibco, Paisley, UK) at a ratio of 0.1 g/mL and incubated at 36.5 ± 0.5 °C for 1, 7, 14, and 21 days to allow potential leaching of ionic species.

Mouse pre-osteoblast MC3T3-E1 cells (ATCC CRL-2593, passages 3–7) were cultured in RPMI-1640 supplemented with 10% fetal bovine serum (FBS; Gibco) and 1% penicillin/streptomycin (Bio-Whittaker Inc., Walkersville, MD, USA) under standard conditions (37 °C, 5% CO_2_, humidified atmosphere). After cell seeding in 96-well plates at a density of 1 × 10^4^ cells/well, cultures were allowed to adhere for 24 h.

Subsequently, 50 µL of serially diluted extract supernatants (1/32, 1/64, and 1/128) were added to each well (final volume: 100 µL). After 24 h of exposure, 20 µL of MTT solution (5 mg/mL in PBS, Sigma, Seoul, Republic of Korea) was added, and plates were incubated for 4 h at 37 °C to allow formazan formation. The resulting crystals were dissolved by adding 100 µL of DMSO (Sigma-Aldrich, St. Louis, MO, USA), followed by 2 h of gentle shaking at room temperature.

Absorbance was measured at 570 nm (reference: 630 nm) using a Benchmark Plus microplate spectrophotometer (Bio-Rad, Hercules, CA, USA), with triplicate measurements for each condition. Cell viability (%) was calculated relative to untreated control cells using the formula:

(2) $$\%\;\mathrm{Cell}\;\mathrm{Viability}\;(\mathrm{CV})\;\;\;\;\;\;\;\;\;CV=\left(\frac{{OD}_{S}}{{OD}_{C}}\right)\times 100$$

(3) $$\%\;\mathrm{Cytotoxicity}\;(\mathrm{CT})\;\;\;\;\;\;\;\;\;\;\;\;CT=\left(\frac{{OD}_{C}-{OD}_{S}}{{OD}_{C}}\right)\times 100$$

where the ${OD}_{C}$ represents the optic density of the control and ${OD}_{S}$ denote the optic density of the samples. The percentage of cell viability for each sample was calculated by taking the control group as 100% [55].

## 3. Results

### 3.1. The Effect of Reaction Time on the HAp Structural Properties

The synthesized powder samples were analyzed by X-ray diffraction (XRD) to assess the influence of hydrothermal reaction time on the crystallographic evolution of hydroxyapatite (HAp). For clarity, the principal diffraction peaks used to distinguish the two phases and their corresponding 2θ values are summarized in Table 1. It is worth noting that reflections between 25° and 50° 2θ, shift slightly to the left in the monoclinic phase with respect to the hexagonal phase, in particular, the (210) plane has a significant shift towards the left. Due to the high structural similarity between the hexagonal and monoclinic HAp polymorphs, phase identification is challenging, as many Bragg reflections overlap or appear at nearly identical 2θ positions [32,36].

**Figure 1:**
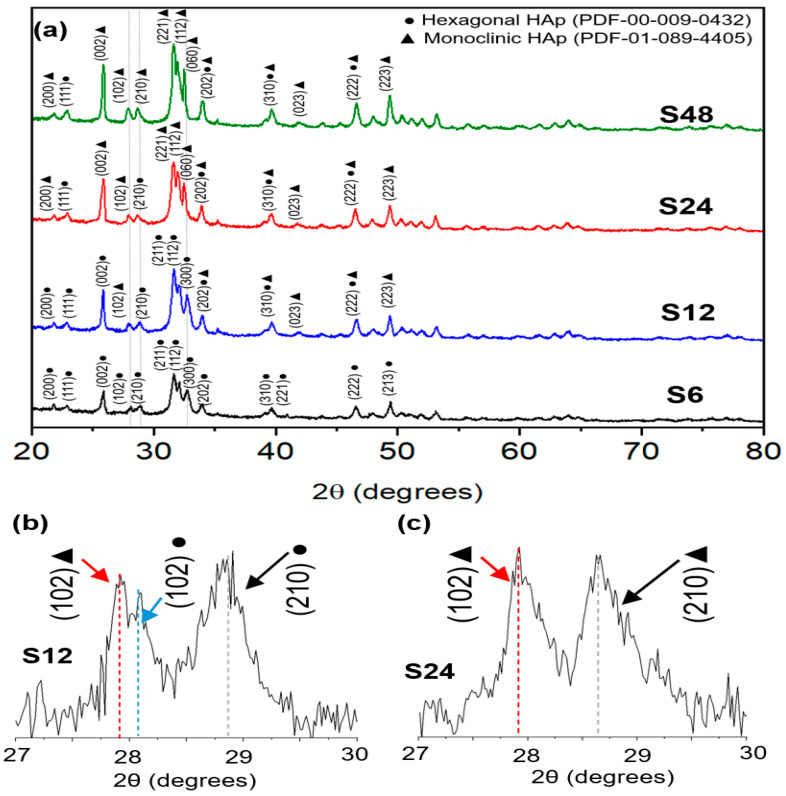
(**a**) X-ray diffraction (XRD) patterns of hydroxyapatite (HAp) nanopowders synthesized at different reaction times (6, 12, 24, and 48 h). The diffractograms show the presence of hexagonal HAp (●) and monoclinic HAp (▲) phases. The sample synthesized at 6 h exhibits only the hexagonal phase, while contributions from the monoclinic phase become evident in samples synthesized for 12 h or longer. (**b**) S12, highlighting the doublet near 28° in the (102) Bragg reflection, indicating the coexistence of hexagonal and monoclinic HAp phases. (**c**) S24, where the doublet disappears and a single reflection at 27.90° appears, assigned to the monoclinic (210) plane.

As shown in Figure 1a, the XRD pattern of the S6 sample exhibits characteristic peaks at 25.88°, 31.78°, 32.19°, 32.91°, and 34.15°, which are assigned to the (002), (211), (112), (300), and (202) planes, respectively, of the hexagonal HAp phase (ICDD PDF No. 00-009-0432). A weak reflection at 40.46°, corresponding to the (221) plane, further supports the assignment to the hexagonal structure.

In the S12 sample, a slight shift in several peaks toward lower 2θ angles is observed, particularly in the 27–30° region, indicating subtle lattice expansion and early-stage structural reorganization. Most notably, the (102) reflection splits into a doublet (Figure 1b), a well-established signature of coexisting hexagonal and monoclinic HAp phases. Additionally, new peaks appear at 40.80° and 49.43°, assigned to the (023) and (223) planes of monoclinic HAp (ICDD PDF No. 01-089-4405), confirming the onset of the phase transition.

For the S24 sample, a distinct low-angle reflection emerges at 21.84°, corresponding to the (200) plan, a hallmark of the monoclinic distortion in HAp. This is accompanied by peaks at 31.75°, 32.18°, and 32.87°, indexed to the (221), (112), and (060) planes of the monoclinic lattice. Notably, the doublet near 28° observed in S12 disappears in S24, replaced by a single sharp peak at 27.90°, assigned to the monoclinic (210) plane (Figure 1c), signaling a progressive dominance of the monoclinic phase.

**Figure 2:**
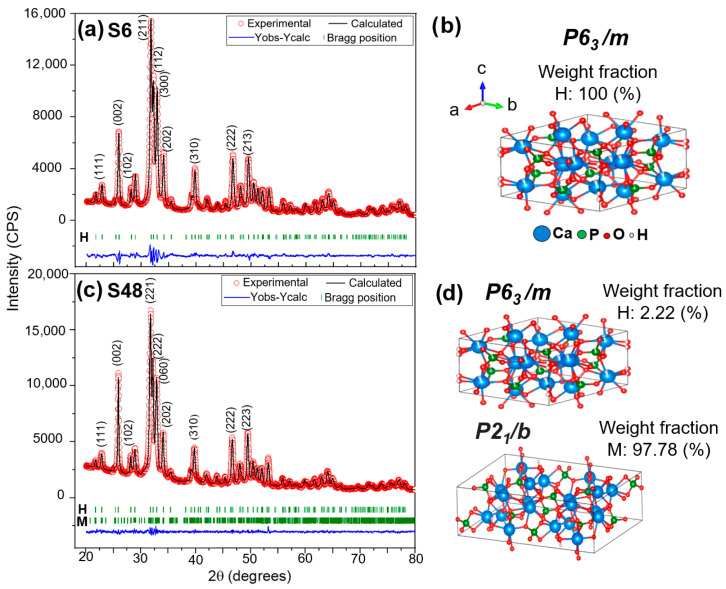
Rietveld refinement and structural models of samples S6 (6 h) and S48 (48 h): (**a**) Rietveld refinement of S6 fitted with a single-phase hexagonal HAp; (**b**) hexagonal unit cell (*P6₃/m*) generated using VESTA; (**c**) Rietveld refinement of S48 fitted with a biphasic hexagonal–monoclinic model; and (**d**) corresponding biphasic unit-cell representation (*P6_3_/m + P2_1_/b*).

Finally, the XRD pattern of the S48 sample (Figure 1a) is predominantly composed of monoclinic Bragg reflections, with minimal residual hexagonal features. This confirms that prolonged hydrothermal treatment promotes the formation of highly monoclinic HAp. Concurrently, a marked increase in diffraction peak intensity is observed with longer reaction times, a trend consistently associated with enhanced crystallinity, as the hydrothermal method facilitates crystal growth and defect annealing [56]. Critically, no secondary calcium phosphate (CaP) phases such as β-tricalcium phosphate (β-TCP, Ca_3_(PO_4_)_2_), tetracalcium phosphate (TTCP, Ca_4_(PO_4_)_2_O), octacalcium phosphate (OCP, Ca_8_(HPO_4_)_2_(PO_4_)_4_·5H_2_O), or dicalcium phosphate dihydrate (DCPD, CaHPO_4_·2H_2_O) were detected in any sample, confirming the phase purity and high crystallinity of the synthesized HAp. Given the extensive peak overlap between the two polymorphs, Rietveld refinement was employed to (i) unambiguously resolve phase coexistence and (ii) quantify the relative fractions of hexagonal and monoclinic HAp as a function of reaction time. Figure 2 displays the Rietveld refinement results for S6 (6 h) and S48 (48 h). The S6 pattern (Figure 2a) is well described by a single-phase hexagonal model (Figure 2b), while S48 (Figure 2c) requires a biphasic model (hexagonal + monoclinic) to achieve a satisfactory fit, as shown in Figure 2d. The refined structural parameters were used to generate the unit-cell representations of both phases using VESTA software, visually illustrating the symmetry differences between P6_3_/m and P2_1_/b HAp.

Table 2 summarizes key parameters obtained from Rietveld refinement, including the goodness-of-fit statistic (χ^2^ = (Rwp/Rexp)^2^), phase composition (%), the c lattice parameter (Å) for each phase, and the average crystallite size. The S6 sample consists exclusively of the hexagonal phase, while S12, S24, and S48 exhibit biphasic mixtures, with the monoclinic fraction increasing progressively with reaction time (10.56%, 73.56%, and 97.78%, respectively). The values remain close to unity and slightly decrease with longer synthesis times, confirming that the structural models provide statistically satisfactory fits to the experimental data.

Rietveld refinement confirms that HAp powders with remarkably high monoclinic phase content were obtained—reaching up to ~98%—using a low-temperature (100 °C) modified hydrothermal method that does not require additional equipment such as microwaves or autoclaves. This exceeds literature values: for instance, sol–gel powders assisted by ultrasonic irradiation yielded ~85% monoclinic phase in Reference [32], while Reference [36] reported 90–95% monoclinic HAp. These results highlight the effectiveness of the modified hydrothermal method in producing highly crystalline monoclinic HAp nanofibers under mild conditions.

Table 2 further shows that, as the monoclinic phase fraction increases, the c-lattice parameter decreases, approaching the characteristic value of the monoclinic phase (c ≈ 6.830 Å). This contraction along the c-axis is associated with the cooperative ordering of OH^−^ groups in the monoclinic structure, which align along the structural channel parallel to the c-direction. Such ordering reduces channel expansion, resulting in a slight shortening of the unit cell along c [16]. Additionally, the average crystallite size decreases with increasing monoclinic content; for example, sample S48 (98% monoclinic phase) exhibited an average crystallite size of approximately 23 nm, a trend consistent with previous reports for HAp nanorods containing 95% monoclinic phase [36].

### 3.2. The Effect of Reaction Time on the HAp Functional Groups

**Figure 3:**
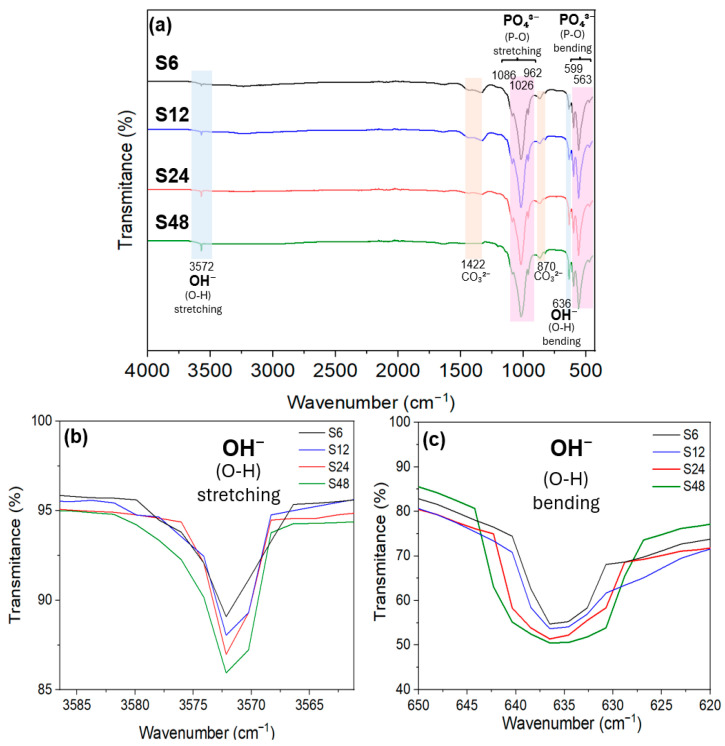
(**a**) FT-IR spectra of HAp powders synthesized at different reaction times, showing the characteristic OH^−^ bands at 3572 and 636 cm^−1^ and the PO_4_^3−^ bands at 563, 599, and 962–1086 cm^−1^. (**b**) Enlarged view of the OH^−^ stretching band. (**c**) Enlarged view of the OH^−^ bending band.

Fourier-transform infrared (FTIR) spectroscopy further corroborates the structural evolution observed by XRD and Rietveld analysis. As shown in Figure 3a, all samples display characteristic vibrational bands of the PO_4_^3−^ group (560–605 cm^−1^ and 1020–1100 cm^−1^) and OH^−^ (stretching at 3572 cm^−1^ and bending at 636 cm^−1^) [57,58]. Weak carbonate bands at 1422 cm^−1^ and 870 cm^−1^, attributed to atmospheric CO_2_ adsorption, diminish with increasing reaction time, indicating enhanced stoichiometry and reduced carbonate incorporation, consistent with the formation of more thermodynamically stable, CO_2_-resistant HAp [59].

Most notably, the intensities of the OH^−^ bands at 636 cm^−1^ and 3572 cm^−1^ increase progressively with longer hydrothermal treatment (Figure 3b,c), while the PO_4_^3−^ bands remain relatively unchanged. This selective enhancement reflects improved ordering and alignment of OH^−^ groups within the HAp lattice, where OH^−^ ions adopt a non-random, cooperative arrangement. The increasing intensity of these modes thus provides complementary spectroscopic evidence for the hexagonal-to-monoclinic phase transition and aligns with the rising monoclinic fraction quantified by Rietveld refinement.

**Figure 4:**
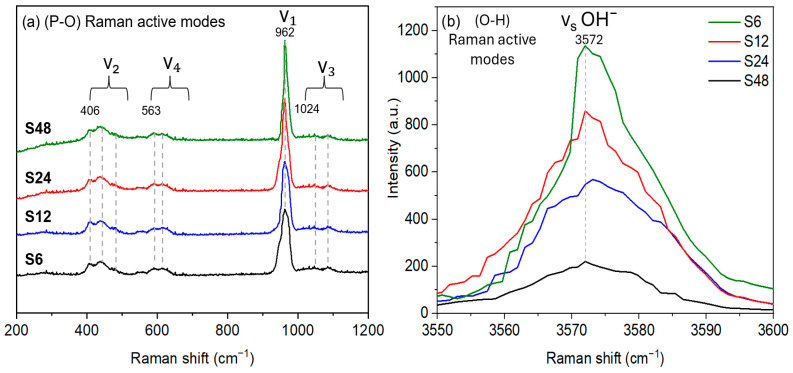
Raman spectra of HAp powders synthesized at different reaction times. (**a**) P–O Raman-active modes (200–1200 cm^−1^, λₑₓₜ = 638 nm). (**b**) OH^−^ band (3550–3600 cm^−1^, λₑₓₜ = 785 nm). The sharpening of the ν_1_(PO_4_^3−^) and νₛ(OH^−^) modes with increasing synthesis time reflects enhanced structural ordering and a higher monoclinic phase fraction.

Figure 4a displays the four characteristic Raman-active vibrational modes of the PO_4_^3−^ group in HAp: the ν_1_ symmetric stretching mode at ~962 cm^−1^, ν_2_ bending at ~406 cm^−1^, ν_3_ asymmetric stretching at ~1024 cm^−1^, and ν_4_ bending at ~563 cm^−1^ [60]. Notably, the ν_1_ band sharpens progressively with increasing hydrothermal time, reflecting enhanced structural ordering and a growing monoclinic phase fraction.

Since the O–H stretching vibration (~3572 cm^−1^) lies outside the PO_4_^3−^ spectral window, its detection serves as a reliable indicator for phase identification [61]. Using a 785 nm near-infrared laser to minimize fluorescence, Raman spectroscopy clearly resolved this OH^−^ mode. As shown in Figure 4b, the intensity of the 3572 cm^−1^ band increases with synthesis time, consistent with FTIR results and further confirming the progressive enrichment of the non-centrosymmetric monoclinic phase.

### 3.3. The Effect of Reaction Time on the HAp Morphology, Size and Composition

**Figure 5:**
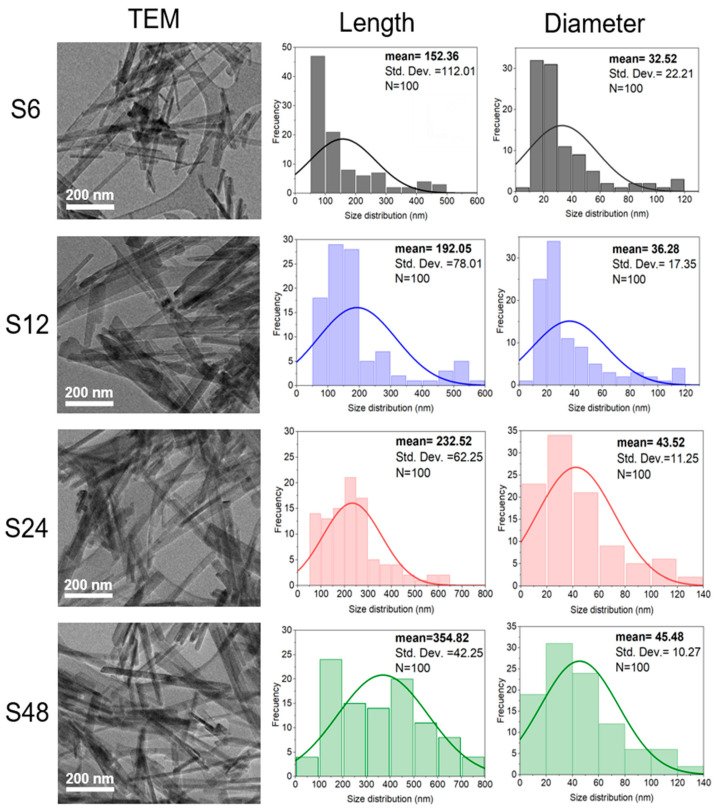
TEM micrographs of HAp powders synthesized at different reaction times (6, 12, 24, and 48 h) and their corresponding length and diameter histograms (TEM scale bar: 200 nm). Regardless of reaction time, all samples display an elongated nanofiber-like morphology.

Transmission electron microscopy (TEM) was employed to assess the morphology, dimensions, and structural features of the HAp nanofibers. As shown in the left column of Figure 5, all samples (regardless of reaction time or phase composition) exhibit well-defined, elongated nanofiber morphologies characteristic of HAp.

Length and diameter distributions, based on 100 manual measurements per sample, are presented in the middle and right columns of Figure 5, respectively, while the nanofiber size ranges, average values (mean ± standard deviation), and aspect ratios (length/diameter, L/D) are summarized in Table 3. S6 sample (6 h) yields nanofibers with lengths in the range of 51.24–483.83 nm and an average length of 152.36 nm, which increases steadily with longer reaction times and higher monoclinic content, reaching 354.82 nm for S48. In contrast, the average diameter shows only a modest increase, from ~33 nm (S6) to 45 nm (S48), with S24 and S48 exhibiting nearly identical diameters (43 and 45 nm, respectively). Likewise, a clear increase in the length-to-diameter ratio (L/D) is observed with increasing reaction time, with L/D values exceeding 1.0, which corresponds to spherical nanoparticles (L = D) [36]. These results indicate that axial elongation is the dominant growth mechanism, while radial growth remains limited under the applied hydrothermal conditions.

**Figure 6:**
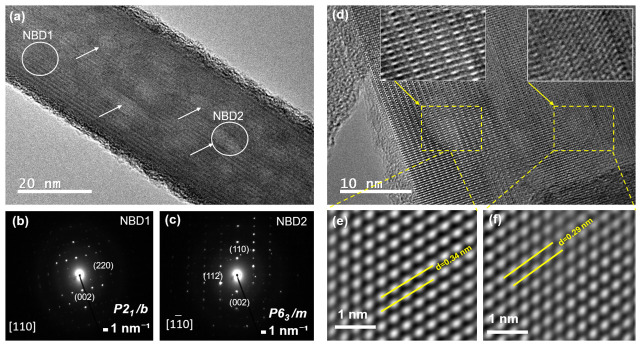
TEM and NBD analysis of HAp nanofibers: (**a**) morphology of S24 showing surface features along the fiber (arrows), suggesting the coexistence of HAp phases (**b**) monoclinic (P2_1_/b) and (**c**) hexagonal (P6_3_/m) NBD patterns; (**d**) HRTEM of S48; (**e**,**f**) FFT-filtered images confirming [002] growth and d-spacings of 0.34 nm (hexagonal) and 0.29 nm (monoclinic).

To probe phase coexistence at the nanoscale, nano-beam electron diffraction (NBD) was performed on an individual nanofiber from S24. Figure 6a reveals surface heterogeneity (arrows), suggesting local phase variations. NBD patterns acquired from the marked regions (Figure 6b,c) confirm this:

Figure 6b (indexed along the [110] zone axis) corresponds to the monoclinic P2_1_/b phase, with growth aligned along the [002] direction (c-axis), consistent with OH^−^ ordering in the structural channel [37]. Figure 6c, in contrast, matches the *hexagonal P6_3_/m phase* [62].

High-resolution TEM (HRTEM) of an S48 nanofiber (Figure 6d) further validates phase purity and orientation. After FFT-based noise filtering, two distinct lattice regions are resolved (Figure 6e,f), both aligned along [002]. Measured interplanar spacings are 0.34 nm (hexagonal (002)) and 0.29 nm (monoclinic (020)), in excellent agreement with simulated structures and literature values [37].

**Figure 7:**
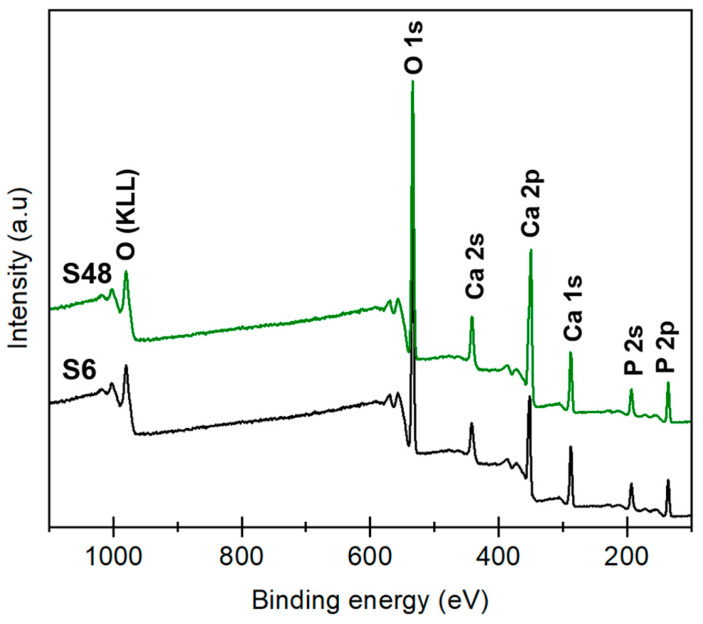
XPS survey spectra for S6 and S48 samples.

Surface composition was analyzed by X-ray photoelectron spectroscopy (XPS). Survey spectra for S6 and S48 (Figure 7) show expected peaks for O 1s (~531 eV), Ca 2p (~347 eV), and P 2p (~133 eV). The Ca 2p intensity is notably higher in S48, consistent with improved stoichiometry.

Table 4 lists the elemental surface composition obtained by XPS alongside the atomic percentages measured by TEM-EDXS. Quantitative analysis (Table 3) reveals that S6 (purely hexagonal) has a surface Ca/P ratio of ~1.60, while S48 achieves ~1.66, approaching the ideal stoichiometric value of 1.67 [63]. This trend, confirmed by both XPS and TEM-EDXS, supports the established correlation between monoclinic ordering and stoichiometry, as Ca-deficient or carbonate-substituted HAp stabilizes the hexagonal phase [24].

**Table 3:** Length/diameter (L/D) and aspect ratio of the HAp nanofibers.

Sample	Length (nm)		Diameter (nm)		Aspect Ratio (L/D)
	Range	Mean ± SD	Range	Mean ± SD	
S6	51.24–483.83	152.36 ± 112.01	11.24–115.93	32.52 ± 22.21	4.69
S12	52.64–587.76	192.05 ± 78.01	12.64–118.80	36.28 ± 17.35	5.29
S24	82.18–608.71	232.52 ± 62.25	18.71–135.65	43.52 ± 11.25	5.34
S48	91.33–788.55	354.82 ± 42.25	19.11–139.65	45.48 ± 10.27	7.80

**Table 4:** Elemental composition obtained by EDXS and XPS.

Sample	Technique	Ca (at.%)	P (at.%)	O (at.%)	Ca/P Ratio
S6	EDXS	23.3	15.0	61.7	1.55
	XPS	23.5	15.0	61.5	1.57
S12	EDXS	24.0	15.4	59.2	1.60
	XPS	24.5	15.2	60.3	1.61
S24	EDXS	25.6	15.5	58.9	1.65
	XPS	25.4	15.4	59.2	1.65
S48	EDXS	25.9	15.6	58.5	1.66
	XPS	25.8	15.5	58.7	1.66

Values are averages of 10 EDXS measurements per sample.

### 3.4. The Effect of Monoclinic HAp on the Piezoelectric Response of Nanofibers

Accurate assessment of piezoelectricity is critical for biomedical applications where electromechanical coupling drives osteogenesis. Resonance tracking piezoresponse force microscopy (RT-PFM), was used to probe local piezoelectric activity, with care taken to mitigate artifacts from electrostatic forces and electrostriction [64]. A robust strategy to distinguish true piezoelectricity involves comparing first-(H1, 1ω) and second-harmonic (H2, 2ω) responses: piezoelectric materials exhibit 1ω > 2ω, whereas electrostriction dominates when 2ω ≥ 1ω [65,66]. Additionally, genuine piezo-ferroelectric systems display a phase contrast of ~90° due to polarization vector rotation [67].

**Figure 8:**
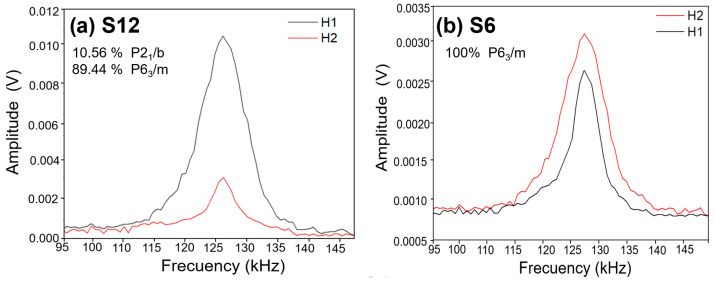
Comparative first-(H1) and second-harmonic (H2) responses obtained from the frequency sweep for samples (**a**) S12 and (**b**) S6. Sample S12, despite its low monoclinic-phase content (10.56%), shows a first-harmonic amplitude five times higher than that of S6, which contains only centrosymmetric hexagonal HAp. In S6, the first-harmonic signal remains below the second, confirming the absence of piezoelectric response in the hexagonal phase.

Figure 8 compares harmonic responses for S6 (hexagonal) and S12 (10.56% monoclinic). Despite its low monoclinic content, S12 shows a first-harmonic amplitude five times greater than S6. Critically, S6 exhibits 2ω > 1ω, confirming the absence of intrinsic piezoelectricity in centrosymmetric hexagonal HAp, consistent with prior reports [32,65].

**Figure 9:**
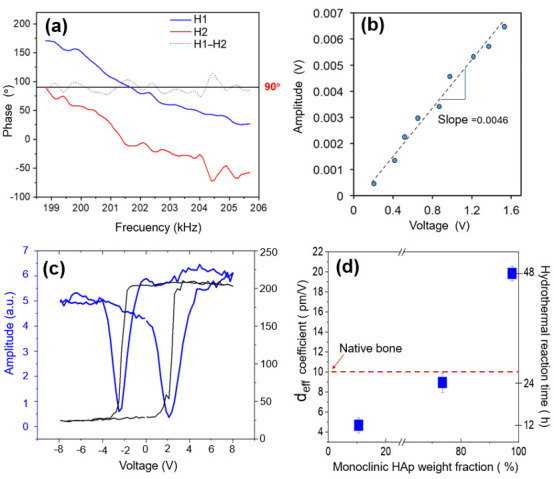
PFM analysis of HAp nanofibers. (**a**) 90° phase difference between the first and second harmonics; (**b**) maximum amplitude as a function of applied voltage (blue line indicates the slope); (**c**) amplitude–voltage and phase–voltage loops measured on a single HAp nanofiber from sample S2; and (**d**) effective piezoelectric coefficient (*d*ₑff) as a function of hydrothermal reaction time and monoclinic HAp phase fraction, compared with the piezoelectric coefficient of native bone.

Figure 9a demonstrates the expected ~90° phase shift between 1ω and 2ω in piezo-ferroelectric materials. Figure 9b shows the linear amplitude–voltage response of an S24 nanofiber, calibrated against a PZT standard using Equation (1). Figure 9c displays a representative amplitude–voltage curve exhibiting the characteristic “butterfly” loop associated with field-induced piezoelectric displacement, providing further evidence of functional non-centrosymmetric ordering in monoclinic HAp. Additionally, the corresponding phase–voltage plot evidences polarization switching, a typical signature of ferroelectric behavior, thereby confirming the piezo-ferroelectric properties of the HAp nanofibers. The resulting effective piezoelectric coefficient (*d*_eff_) measured in triplicate for each powder sample, increases with monoclinic content, yielding average values ± standard deviations of 4.68 ± 1.12 pm/V for S12, 8.95 ± 1.89 pm/V for S24, and 19.85 ± 1.15 pm/V for S48, respectively. These values (Figure 9d) correlate directly with monoclinic fraction. Notably, S24 matches the piezoelectric response of native bone (~10 pm/V) [9,10], suggesting strong potential for bone-regeneration scaffolds. The S48 nanofibers, containing ~98% monoclinic phase, exhibit a two-fold enhancement over bone-like performance, highlighting their suitability for applications requiring high electromechanical coupling, such as stimuli-responsive implants or piezoelectric tissue engineering matrices [68].

Piezoresponse force microscopy (PFM) is a powerful technique for investigating the behavior of ferroelectric domains, providing simultaneous information on surface topography and electromechanical response. In the PFM images, amplitude maps indicate the strength of the local electromechanical coupling and highlight ferroelectric domain boundaries, while the contrast in the phase maps reveals differences in polarization orientation between adjacent domains, confirming the intrinsic piezo-ferroelectric nature of the material [66].

**Figure 10:**
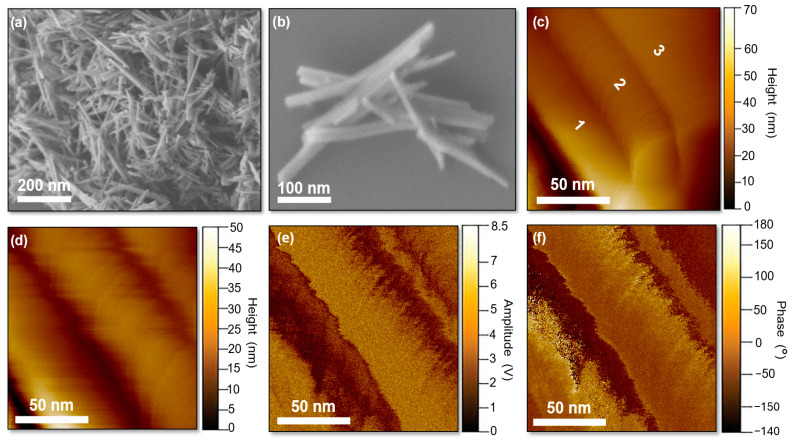
(**a**,**b**) SEM images of clustered HAp nanofibers selected for PFM analysis. (**c**,**d**) AFM topography images of interconnected and individual S48 nanofibers (<50 nm diameter) labeled as 1, 2 and 3. (**e**) PFM amplitude and (**f**) phase maps of the same region showing ferroelectric-like domain contrast. Amplitude highlights domain boundaries, while phase reflects polarization orientation; both are independent of topography, confirming the electromechanical coupling of the HAp nanofibers.

In this work, PFM images are presented, in contrast to several previous studies that report only amplitude–voltage curves. Figure 10 shows the PFM images obtained for the HAp nanofibers. Figure 10a,b present SEM images illustrating the sample surface analyzed by PFM, where clusters of overlapping HAp nanofibers can be observed. Figure 10c displays an AFM topography image of three interconnected nanofibers, with a thickness below 50 nm identified for the nanofiber labeled as number 2. Figure 10d presents a topography image of individual S48 HAp nanofibers with diameters below 50 nm, consistent with the TEM results. The corresponding PFM amplitude and phase maps (Figure 10e,f) reveal ferroelectric-like domain contrast: domain boundaries are visible in the amplitude map, while ferroelectric- like domains are clearly resolved in the PFM phase map. Overall, these findings provide clear evidence of the piezo-ferroelectric properties of the HAp nanofibers synthesized in this work.

### 3.5. The Effect of Monoclinic Phase on the Biocompatibility of the HAp Nanofibers

**Figure 11:**
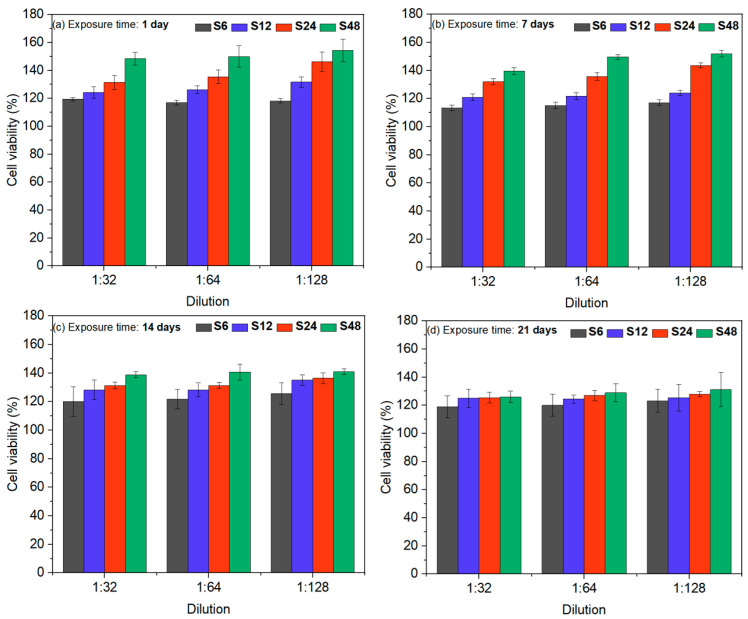
Cell viability of MC3T3-E1 preosteoblasts exposed to ion-release extracts of HAp powders at different dilution levels for (**a**) 1, (**b**) 7, (**c**) 14, and (**d**) 21 days. By day 21, all powders showed stabilized and comparable biocompatibility regardless of their hexagonal-to-monoclinic phase ratios.

**Figure 12:**
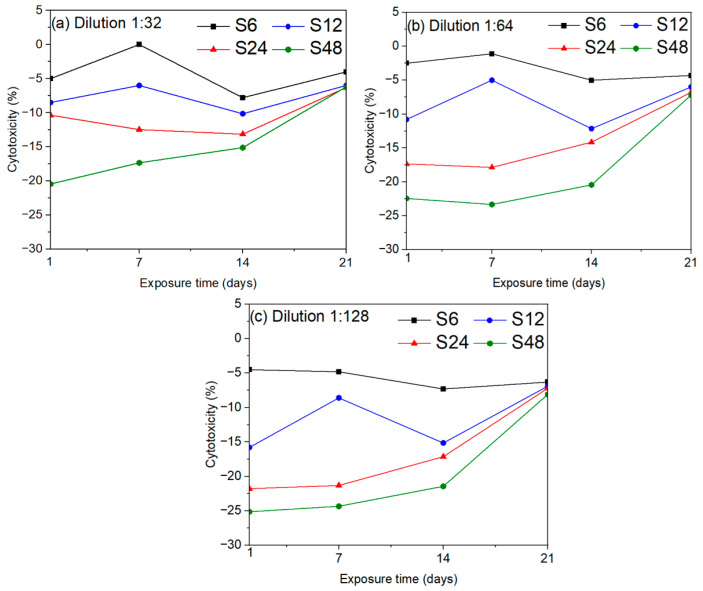
Cytotoxicity of HAp nanofibers at different exposure times (1–21 days) and dilutions: (**a**) 1:31, (**b**) 1:64, and (**c**) 1:128, as determined by the MTT assay. All samples stabilize below 6% cytotoxicity after 21 days, remaining within the non-cytotoxic range defined by ISO 10993 [54] and ASTM F895 [69].

Figure 11 and Figure 12 show the percentages of cell viability and the corresponding cytotoxicity, respectively, derived from UV–Vis spectroscopy using Equations (2) and (3) based on the results of the in vitro MTT assay. Figure 11 demonstrates that all HAp powders—regardless of ion-release exposure time and dilution—exhibit cell viability values above 100%, confirming their biocompatible nature. A more detailed analysis of Figure 11 reveals that, at early time points (1 and 7 days), samples S24 and S48—characterized by higher proportions of the monoclinic, osteoconductive, and piezoelectric phase (97.78% and 73.56%, respectively)—display enhanced cellular activity compared to both the pure hexagonal HA sample (S6) and sample S12 (10.54% monoclinic phase), particularly at higher dilutions. This increased early activity may be attributed to the larger fraction of the monoclinic phase, which can promote initial cell adhesion and proliferation.

In Figure 11c, the differences in cellular activity between the pure hexagonal HAp powder (S6) and the powders containing the monoclinic phase gradually diminish, indicating a convergence in their biological behavior over time. Figure 11d shows that cell viability eventually stabilizes across all samples. At this stage, cell–HAp interactions remain consistent regardless of the relative proportions of hexagonal or monoclinic phases, and all groups exhibit comparable biocompatibility after prolonged exposure.

Figure 12 presents the cytotoxicity values obtained from the MTT assay at different exposure times (1, 7, 14, and 21 days) and dilution levels (1:31, 1:64, and 1:128). Consistent with cell viability results, samples with the highest monoclinic phase content (S48 and S24) exhibit enhanced cellular activity and lower cytotoxicity at short exposure times, especially at the highest dilution (1:128), as shown in Figure 12c. Moreover, regardless of synthesis time or the relative proportions of hexagonal and monoclinic phases, the cytotoxicity of the HAp nanofibers stabilizes below 6% after 21 days of exposure. All evaluated samples fall within the non-cytotoxic range, meeting the requirements of ISO 10993 [54] and ASTM F895 [69], which define acceptable cytotoxicity as total cytotoxicity (TC) values below 10%.

## 4. Discussion

According to the physicochemical characterization of the hydroxyapatite (HAp) powders, reaction time is a critical parameter governing the phase transition from hexagonal to monoclinic HAp. To the best of the authors’ knowledge, this is the first report of highly crystalline, stoichiometric (Ca/P ≈ 1.67), elongated monoclinic HAp nanofibers synthesized via a modified low-temperature hydrothermal method, without the use of an autoclave or microwave assistance.

This systematic study elucidates the pivotal role of synthesis duration in tuning the physicochemical properties of HAp, enabling precise control over its morphology, size, shape, and piezoelectric response. Rietveld refinement confirmed that the monoclinic phase dominates (~98%) in samples synthesized for 48 h. Notably, the crystallite size of this phase decreased from approximately 39 nm to 23 nm as the monoclinic fraction increased. In contrast, the hexagonal phase exhibited the opposite trend: its crystallite size grew from 23 nm to 38 nm with prolonged reaction time, consistent with previous literature [36].

In this work, the hexagonal HAp phase remained stable after 6 h of reaction. However, coexistence of both hexagonal and monoclinic phases became detectable (10.56%) from 12 h onward. Several studies have shown that the monoclinic HAp phase (space group P2_1_/b) can be regarded as a superstructure derived from the conventional hexagonal phase. This transformation arises from the cooperative ordering of hydroxyl (OH^−^) columns aligned along the c-axis channel, columns that exhibit significant orientational disorder in the hexagonal arrangement [25]. When OH^−^ groups become ordered, hexagonal symmetry is broken, giving rise to the monoclinic unit cell.

Consequently, the symmetric stretching vibration of OH^−^ (νₛ(OH^−^)) at ~3572 cm^−1^ appears more intense in monoclinic HAp due to its fully ordered and aligned OH^−^ arrangement, lower crystallographic symmetry, and less distorted anionic channels, all of which enhance the infrared activity of this vibrational mode. In contrast, partial disorder in the hexagonal phase markedly reduces this band’s intensity. This structural ordering also leads to a more stable and coherent lattice, which explains the sharpening of Raman bands (particularly the ν_1_ symmetric stretching mode) and the enhanced intensity of OH^−^-related vibrational features [18].

Thus, in this study, the sharpening of the Raman ν_1_ band is directly linked to higher crystallinity, reduced local disorder, and more well-defined vibrational behavior, providing clear spectroscopic evidence of the monoclinic phase. An additional indicator of this phase is the slight contraction along the c-axis, consistent with the cooperative ordering of OH^−^ groups in the monoclinic structure. Unlike the partially disordered OH^−^ dipoles in the hexagonal phase, the ordered alignment in monoclinic HAp reduces expansion of the structural channels along the c-axis, resulting in a smaller c lattice parameter [29,33].

Rietveld refinement revealed a reduced c parameter of 6.8289 Å for the sample with the highest monoclinic content, a value in excellent agreement with the theoretical c parameter of 6.83 Å for monoclinic HAp [23].

For the bone tissue engineering (BTE) field, both the piezoelectric monoclinic HAp phase and elongated nanostructures (such as nanorods, nanofibers, nanobelts, and nanowires) are highly desirable. Their enhanced osteogenic performance stems from increased surface area, which promotes stronger interactions with osteoblasts on scaffolds [13]. TEM analysis confirmed that the synthesis protocol consistently produced well-defined elongated nanofibers across all reaction times, irrespective of the hexagonal-to-monoclinic phase ratio. Moreover, nanofiber dimensions were time-dependent: the 48 h sample exhibited an average length of 354.82 nm and a diameter of 45 nm.

Although monoclinic HAp has been reported previously [22,32,36], a comprehensive study correlating synthesis time with phase evolution, morphology, piezoelectric response, and biological relevance has been lacking. In the present work not only demonstrates tunable hexagonal-to-monoclinic phase transition through reaction time, supported by extensive structural characterization, but also includes direct evaluation of the piezoelectric response via room-temperature by RT-PFM. Remarkably, effective piezoelectric coefficients (*d*_eff_) were measured directly on HAp powders, a significant advance, as such measurements are typically limited to bulk ceramics or thin films. The S24 sample (73.54% monoclinic HAp) yielded a deff of 8.95 pm/V, approaching the piezoelectric coefficient of native bone (~10 pm/V), indicating strong potential for bone regeneration. Even more notably, the S48 nanofibers (~98% monoclinic HAp) exhibited a markedly enhanced piezoelectric response, with *d*_eff_ = 19.85 pm/V, positioning them as promising candidates for applications requiring robust electromechanical coupling [70].

The comprehensive characterization of the HAp nanofibers suggests that their enhanced piezoelectric response arises from the combined effects of high monoclinic fraction, high crystallinity, and elongated morphology. The monoclinic P2_1_/b phase provides a non-centrosymmetric structure favorable for polarization, while high crystallinity reduces domain pinning and minimizes point defects. The elongated nanofiber shape (high L/D ratio) promotes preferential growth along the HAp c-axis and axial dipole alignment, further enhancing the local piezoelectric response. In contrast, bulk HAp generally exhibits lower piezoelectric coefficients due to structural defects or porosity, which can lead to leakage currents or domain cancelation. These results highlight the critical role of phase purity, structural order, and fiber morphology in maximizing nanoscale piezoelectricity.

Finally, in vitro MTT assays confirmed that the monoclinic phase does not compromise biocompatibility: all samples met the cytocompatibility requirements of ISO 10993 [54] and ASTM F895 [69]. Owing to their combined piezoelectric and biological properties, the HAp nanofibers developed in this work hold promise not only for bone regeneration scaffolds [26] but also for diverse applications such as protein functionalization [71], drug delivery [72], gas sensing [68], catalysis [28], and water remediation [73].

## 5. Conclusions

This study demonstrates that synthesis time critically controls the hexagonal-to-monoclinic phase transition in HAp nanofibers prepared via a modified low-temperature hydrothermal method at 100 °C without an autoclave, distinguishing it from conventional approaches. For the first time, highly crystalline, stoichiometric monoclinic HAp nanofibers were obtained under these mild conditions, with no detectable secondary calcium phosphate (CaP) phases.

Increasing the reaction time promoted higher monoclinic phase content, improved stoichiometry, and greater nanofiber length. Crucially, the rise in monoclinic fraction enhanced piezoelectric performance without impairing biocompatibility. These findings confirm the stability of the monoclinic phase and demonstrate that its coexistence with the hexagonal phase can be precisely modulated via hydrothermal synthesis to tailor the electromechanical properties of HAp nanofibers for bone regeneration and other advanced applications.

## Figures and Tables

**Table 1:** Main planes and angles of the H-HAp and HM-HAp samples.

Hexagonal (*P6_3_/m)* (PDF-00-009-0432)		Monoclinic (*P2_1_/b)* (PDF-01-089-4405)	
Plane	Peak	Plane	Peak
(200)	21.783°	(200)	21.821°
(002)	25.880°	(002)	25.853°
(102)	28.131°	(102)	28.102°
(210)	28.851°	(210)	28.123°
(211)	31.785°	(221)	31.740°
(112)	32.201°	(112)	32.185°
(300)	32.921°	(060)	32.872°
(213)	49.499°	(223)	49.437°

**Table 2:** Obtained phases, lattice parameters and crystallite size by Rietveld analysis.

	Goodness-of-Fit			Hexagonal HAp (*P6_3_/m*)			Monoclinic HAp (*P2_1_/b*)		
Sample	R_wp_	R_exp_	χ^2^	Lattice Parameter *c* (Å)	Phase (%)	Crystallite Size (nm)	Lattice Parameter *c* (Å)	Phase (%)	Crystallite Size (nm)
S6	7.89	7.35	1.15	6.8767	100	23.16	-	-	-
S12	7.25	6.68	1.18	6.8623	89.44	19.14	6.8567	10.56	39.12
S24	6.39	6.15	1.13	6.8456	26.44	22.15	6.8313	73.56	28.15
S48	7.29	7.05	1.06	6.8392	2.22	38.24	6.8289	97.78	23.21

## Data Availability

The original contributions presented in this study are included in the article. Further inquiries can be directed to the corresponding author.
